# Beyond Surface Area: The Role of Carrier Media Structure on Nitrification Performance and Biomass Activity in MBBR Systems Under High Nitrogen Loads

**DOI:** 10.1002/wer.70495

**Published:** 2026-07-16

**Authors:** Isabelli Dias Bassin, Renato Rocha Valerio, João Paulo Bassin

**Affiliations:** ^1^ Department of Biochemical Engineering, School of Chemistry Federal University of Rio de Janeiro Rio de Janeiro Brazil; ^2^ Chemical Engineering Program, COPPE Federal University of Rio de Janeiro Rio de Janeiro Brazil; ^3^ Civil Engineering Program, COPPE Federal University of Rio de Janeiro Rio de Janeiro Brazil

**Keywords:** biofilm stability, biomass retention, carrier media structure, moving bed biofilm reactor, nitrogen loading rate

## Abstract

This study evaluated the effects of increasing nitrogen loading rates and carrier type on nitrification performance and biomass characteristics in moving bed biofilm reactors (MBBRs) operated under autotrophic conditions. Two laboratory‐scale reactors (R1 and R2), operated for over 1200 days and filled with distinct carrier media, non‐porous Kaldnes K1 and porous Mutag Biochip, were subjected to stepwise nitrogen loading up to 11 g NH_4_^+^‐N/(m^2^.d). Both systems maintained stable ammonium removal up to 3 g NH_4_^+^‐N/(m^2^.d), but higher loading rates led to distinct performance patterns. R1, filled with Kaldnes K1 media, exhibited greater stability and higher nitrogen removal rates (exceeding 6 g NH_4_^+^‐N/(m^2^.d)), whereas R2 (filled with Mutag Biochip carriers) showed reduced efficiency and higher nitrite accumulation above 6 g NH_4_^+^‐N/(m^2^.d), likely associated with free ammonia inhibition and biomass detachment. Biomass accumulation was assessed through measurements of attached solids and microscopic examination of the carrier media. Although both reactors had the same theoretical surface area, R1 developed a thicker and more stable biofilm with higher biomass retention, whereas R2 showed limited immobilized biomass accumulation. The results suggest that nitrification efficiency under high nitrogen loads was influenced not only by available surface area and chemical inhibition, but also by the structural characteristics of the carrier, which affected biofilm accumulation and mass transfer. These findings provide insights that may support carrier selection and operational strategies to enhance the stability and performance of MBBRs treating high‐strength nitrogen wastewaters.

## Abstract (highlights)

**Summary**

- Carrier features strongly influence biofilm stability and nitrification performance under high ammonium loading conditions.
- Non‐porous carriers promote higher biomass retention and greater process stability in autotrophic MBBR systems.
- Porous carriers are more susceptible to biofilm detachment and nitrite accumulation at elevated nitrogen loads.
- Long‐term operation reveals that theoretical surface area alone is insufficient for predicting nitrification capacity.

## Graphical abstract

Carrier structural configuration, rather than nominal surface area alone, influenced nitrification performance in long‐term autotrophic MBBRs operated under high nitrogen loads. Non‐porous media promoted greater biofilm accumulation and more stable ammonium removal, suggesting that biomass retention capacity plays an important role in sustaining nitrification under elevated loading conditions.

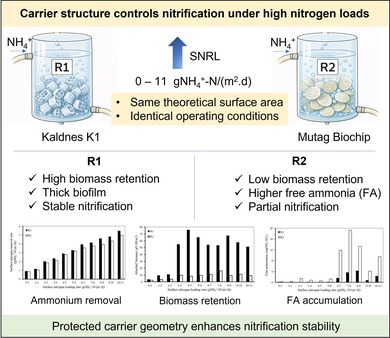

## 1 Introduction

The discharge of ammonium‐rich wastewaters is a major environmental concern due to eutrophication and aquatic toxicity. High ammonium concentrations, which may reach up to 5000 mg NH_4_^+^/L, are commonly found in anaerobic digester effluents, landfill leachate, livestock wastewater, and certain industrial streams, requiring robust treatment processes capable of handling elevated nitrogen loads (Bassin 2018; Liu et al. 2019).

Biological wastewater treatment processes are widely applied for nitrogen removal, with nitrification representing the critical and often rate‐limiting step due to the slow growth and sensitivity of autotrophic nitrifiers. Conventional activated sludge (CAS) systems are usually constrained by operational limitations, including poor sludge settleability that can cause solids washout and hinder the retention of slow‐growing nitrifying bacteria (Güneş et al. 2019; Leyva‐Díaz et al. 2020). In contrast, immobilized biomass systems, also referred to as biofilm reactors, provide improved operational stability, resilience to load fluctuations, and biomass retention that support the growth and activity of slow‐growing nitrifying bacteria (Jahren et al. 2002; Jamal Khan et al. 2011; Bassin et al. 2012; Ali et al. 2025).

Among immobilized biomass systems, the moving bed biofilm reactor (MBBR) is widely used to enhance nitrification, particularly in overloaded CAS systems (Hem et al. 1994; Ødegaard 2006; Deena et al. 2022; Nourredine and Barjenbruch 2024). In MBBRs, biofilm grows on suspended carriers, enabling high biomass retention and stable microbial activity (Bassin 2018). Laboratory, pilot, and full‐scale studies have demonstrated their effectiveness for organic matter and nitrogen removal (Bassin et al. 2016; Lima et al. 2016; Zheng et al. 2019).

Nitrification performance in MBBRs is strongly influenced by operational factors, such as organic and nitrogen loading rates. When applied as a secondary and tertiary treatment step for relatively low‐nitrogen wastewaters, such as domestic sewage, MBBRs can efficiently remove ammonium, with biofilm composition and structure governed mainly by the availability of nitrogenous substrates (Zhang et al. 2013; Ashkanani et al. 2019). However, under high nitrogen loading, nitrification performance may be substantially impaired due to the effects posed by inhibitors, such as free ammonia (FA) and free nitrous acid (FNA), on ammonia‐oxidizing bacteria (AOB) and nitrite‐oxidizing bacteria (NOB) (Liu et al. 2019; Prosser 1989; Vadivelu et al. 2006; Ren et al. 2024). Additionally, excessive biofilm growth under these conditions increases the risk of carrier clogging, further limiting reactor efficiency and reducing overall nitrification rates (Forrest et al. 2016; Young et al. 2016). Data from studies on MBBRs reporting surface nitrogen loading and corresponding removal rates under different influent organic matter conditions are shown in Table S1.

Although several studies indicate that the applied nitrogen load is often a dominant factor governing nitrification performance in MBBR systems, another critical aspect relates to the characteristics of the carrier media used for microorganism immobilization. Over the years, numerous carriers manufactured from different materials, such as polyethylene, polypropylene, polyvinyl alcohol (PVA), polyurethane foam, and other synthetic polymers, have been developed for MBBR applications (Ødegaard et al. 2000; Bassin 2018; Deena et al. 2022). These carriers differ not only in material composition but also in geometry, density, porosity, and protected surface area. Such characteristics influence mass transfer, biomass attachment and detachment patterns, biofilm thickness, and microbial stratification (Ødegaard et al. 2000; Forrest et al. 2016; Young et al. 2016; Deena et al. 2022; Nayeri et al. 2025). These factors have also been recognized as important for the stable operation of nitrogen‐removal biofilm systems, including deammonification MBBRs (Zekker et al. 2013). Additionally, the distribution of both adhered and suspended biomass within the reactor is influenced by media properties (Bassin et al. 2016; Ødegaard et al. 2000).

Consequently, beyond nominal specific surface area, carrier structural properties may significantly affect nitrification stability, maximum attainable loading rates, and long‐term operational performance, particularly under extreme nitrogen loads. Studies addressing the effect of media carriers on nitrification under autotrophic conditions are particularly important to exclude heterotrophic interference, and because many wastewaters, such as anaerobic digester effluents, landfill leachate, livestock manure wastewater, and certain industrial streams, are rich in nitrogen (Bassin 2018). Levstek and Plazl (2009) evaluated two MBBR carriers in the absence of external COD: a polyethylene media (Kaldnes K1) and PVA gel beads. They observed that both led to similar maximum nitrification rates (≈49–50 mg NH_4_^+^‐N/(L.h)), despite the much higher effective specific surface area of PVA media. Forrest et al. (2016) investigated tertiary nitrifying MBBRs using three carrier types to assess ammonium removal, biofilm morphology, and solids production. They found that, under moderate nitrogen loads, carrier type did not significantly affect nitrification performance. However, higher surface area media were more susceptible to clogging at elevated load conditions and showed slower recovery thereafter. Jang et al. (2023) compared two MBBR carriers treating nitrogen‐rich anaerobic digestion effluent. At a high HRT (20 days), the two tested carriers allowed achieving almost complete ammonium removal. However, upon HRT reduction up to 10 days, only the reactor filled with high‐density polyethylene Mutag Biochip carriers maintained high ammonium removal. In contrast, the other containing fiber ball media exhibited partial nitrification. The superior performance was linked to higher abundance and diversity of nitrifying bacteria in the biochip media.

Despite the widespread application of MBBRs, long‐term studies systematically evaluating their operational limits under progressively increasing ammonium loads and strictly autotrophic conditions remain scarce. Most available research relies on short‐term experiments or moderate nitrogen loading, providing limited insight into system resilience under more extreme conditions. Furthermore, quantitative information on maximum nitrification capacity in the absence of external COD across different influent nitrogen levels and its relationship with carrier type is still limited (Janka et al. 2025). It should also be noted that most commonly applied carriers are non‐porous, whereas porous media exhibit different mass‐transfer and biofilm retention dynamics, potentially leading to distinct nitrification behavior (di Biase et al. 2019).

Therefore, this study presents a long‐term (over 1200 days) comparative assessment of two MBBR systems operated under strictly autotrophic conditions. The reactors were filled with carriers exhibiting different structural characteristics (non‐porous and porous), yet designed to provide the same theoretical surface area. Both reactors were subjected to stepwise increases in surface nitrogen loading up to 11 g NH_4_^+^‐N/(m^2^.d) to investigate how differences in carrier structure may influence the operational limits of nitrification and biofilm accumulation, as well as the interplay between apparent biomass‐specific nitrifying activity, attached and suspended biomass distribution, and reactor stability.

## 2 Materials and Methods

### 2.1 Experimental Apparatus and Reactor Operating Conditions

The experiments were performed in two laboratory‐scale MBBR systems (R1 and R2), each with a working volume of 0.2 L, operated in continuous mode for over 1200 days. R1 was filled with Kaldnes K1 nonporous plastic carriers (500 m^2^/m^3^), while R2 contained porous Mutag Biochip carriers with a surface area of 3000 m^2^/m^3^. Further details regarding carrier geometry, dimensions, filling fractions, and number of carrier units are provided in Table S2. The filling fractions were set at 50% for R1 and 8.3% for R2 to ensure that both reactors operated with the same specific surface area (250 m^2^/m^3^). Considering the reactor volumes, the total surface area available for biofilm growth was 0.05 m^2^ in each system. To achieve the desired filling fraction in each reactor, approximately 100 and 29 carrier units were used in R1 and R2, respectively. The experimental setup was designed to enable a controlled comparative evaluation of the two carrier media under identical hydrodynamic and operational conditions. Therefore, the influence of carrier configuration on biomass retention and nitrification performance under progressively increasing nitrogen loading conditions during long‐term operation could be comparatively assessed.

Throughout the study, the reactors were operated under 11 experimental conditions (Table 1), in which the nitrogen loading rate was gradually increased while maintaining a constant hydraulic retention time (HRT) of 3 h, in order to evaluate its impact on nitrification performance and determine the maximum ammonium load that could be removed. The duration of each operational condition was selected to allow reactor adaptation and stabilization after each increase in SNLR, considering the relatively slow growth kinetics of nitrifying microorganisms.

**TABLE 1:** Operating conditions of the MBBR systems over the experimental runs.

Run	Applied SNLR (gNH_4_^+^‐N/(m^2^.d))	Applied VNLR (kgNH_4_^+^‐N/(m^3^.d))	Operating days	Time of operation (days)
1	0–1	0–0.25	1–12	12
2	1–2	0.25–0.5	13–107	94
3	2–3	0.5–0.75	108–253	145
4	3–4	0.75–1.0	254–289	35
5	4–5	1.0–1.25	290–428	138
6	5–6	1.25–1.5	429–563	135
7	6–7	1.5–1.75	564–666	102
8	7–8	1.75–2.0	667–913	246
9	8–9	2.0–2.25	914–1106	192
10	9–10	2.25–2.5	1107–1173	66
11	10–11	2.5–2.75	1174–1220	46

Both reactors were inoculated with activated sludge from a municipal wastewater treatment plant (Rio de Janeiro, Brazil) designed for COD removal and nitrification, ensuring an initial microbial community capable of sustaining autotrophic nitrogen conversions. The total inoculum volume was 100 mL, applied in two equal portions of 50 mL during the first and second weeks of operation. During this initial start‐up period, effluent biomass was reintroduced into the reactors to promote biofilm establishment and enhance nitrifying biomass retention.

The reactors were fed with laboratory‐prepared synthetic wastewater to maintain precise control over substrate concentrations. Ammonium chloride (NH_4_Cl) and sodium bicarbonate (NaHCO_3_) served as the nitrogen and inorganic carbon (alkalinity) sources, respectively. The synthetic wastewater was prepared in distilled water with the following composition: 108 mg/L NH_4_Cl (yielding around 30 mg NH_4_^+^‐N/L in the influent), 200 mg/L NaHCO_3_, 111 mg/L NaCl, 27 mg/L MgSO_4_, 27 mg/L KH_2_PO_4_, and 40.5 mg/L K_2_HPO_4_. A trace element solution, as described by Bassin et al. (2012), was added at 0.5 mL/L of wastewater prepared. The synthetic wastewater was stored at 4°C to prevent degradation under ambient conditions and maintained at a pH of 7.5–8.0.

Throughout the study, the composition of the synthetic wastewater was adjusted to achieve ammonium nitrogen loads ranging from 0.9 to 11 g NH_4_^+^‐N/(m^2^.d) (Table 1), while maintaining a bicarbonate‐to‐ammonium nitrogen ratio of approximately 7 to ensure sufficient inorganic carbon for complete nitrification (Wang et al. 2023) and keeping all other constituents at constant concentrations. This controlled approach enabled a systematic evaluation of the effect of increasing nitrogen load on nitrification performance and biomass response.

Oxygen supply and mixing were achieved by introducing air into the reactors through diffusers positioned at the bottom of each tank. The airflow was maintained at approximately 4 L/min to ensure uniform hydraulic conditions and continuous suspension of the carriers while providing adequate oxygen transfer to the bulk liquid. Dissolved oxygen (DO) concentrations were consistently maintained between 4 and 5 mg/L throughout the operation of the reactors. Ambient temperature was approximately 22 ± 4°C using air conditioning, and pH was controlled within the range of 6.8–7.5 using 1 M NaOH or 1 M HCl. DO and pH were periodically measured using previously calibrated probes.

### 2.2 Nitrifying Activity Assessment and Calculation Procedures

A nitrogen mass balance was established under steady‐state conditions by comparing influent nitrogen, supplied exclusively as ammonium, with effluent inorganic nitrogen species, including residual ammonium and nitrite and nitrate generated through nitrification. Because no external organic carbon source was provided and no intentional anoxic zones were established, biological nitrogen removal via denitrification was not expected to occur to a significant extent.

Soluble nitrogen conservation was defined as the fraction of influent ammonium recovered in the effluent as oxidized nitrogen species (NO_2_^−^ and NO_3_^−^). Total nitrogen removal from the liquid phase was calculated as the difference between influent ammonium nitrogen and the total measured soluble inorganic nitrogen in the effluent. Any discrepancy was attributed primarily to nitrogen assimilation into biomass and, potentially, to minor gaseous losses associated with nitrification under elevated loading conditions.

The concentration of non‐ionized species, particularly FA and FNA, was estimated due to their recognized inhibitory effects on AOB and NOB, particularly under elevated nitrogen loading conditions and pH variations. FA and FNA were calculated based on the equilibrium relationships described in the Supporting Information, using the measured values of pH, temperature, and the concentrations of ammonium and nitrite, respectively.

### 2.3 Analytical Methods

Ammonium, total suspended solids (TSS), and volatile suspended solids (VSS) were measured according to Standard Methods (APHA 2005). Nitrite and nitrate concentrations were determined using commercial analytical kits (Hach Co., Loveland, Colorado, USA). All analyses were performed in triplicate, and average values are presented. For each SNLR range, performance metrics were expressed as average values over the corresponding operational period. pH was measured using a Hanna Instruments HI 2221 electrode. To examine biomass attached to the carriers, two media were sampled from each reactor on three occasions during the final operating period. Only two carriers were removed at each sampling event to minimize disturbances to reactor operation caused by biomass removal. After analysis, the sampled carriers were replaced with new clean carriers to maintain the original filling fraction and the same surface area in the reactors. The collected carriers were photographed using a Zeiss Axio Zoom. V16 microscope camera to visualize biofilm structure and morphology.

The concentration of biomass attached to the carriers, expressed as total attached solids (TAS) and volatile attached solids (VAS), was determined following protocols developed in our previous studies (Bassin et al. 2016; Fonseca and Bassin 2019), with minor modifications. For the non‐porous Kaldnes K1 carriers, biofilm detachment is relatively straightforward. Three representative carriers were collected, placed in Falcon tubes, and treated with a known volume of 1 N NaOH to facilitate biofilm removal. The biomass released into the solution was then quantified using the same procedure applied for suspended solids determination (APHA 2005). Any residual NaOH solids were subtracted from the measured solids to obtain the net biomass content. To extrapolate these measurements to the entire reactor, both the total number of carriers and the reactor volume were considered. The sum of VAS and VSS was defined as the volatile total solids (VTS) in the system. On the other hand, biomass extraction from the porous Mutag Biochip carriers is challenging due to microbial growth within the pores. Therefore, a direct extraction method was not used to determine adhered solids. Instead, three representative carriers were dried in an oven at 105°C for 4 h and subsequently weighed. Total adhered solids per carrier (TAS, g/carrier) were calculated by subtracting the average mass of clean, dry carriers (without biofilm) from the measured mass of carriers with biofilm. To estimate total adhered solids per reactor volume (g/L), the total number of carriers (n) in the reactor and its volume were considered.

## 3 Results

### 3.1 General Description of Reactor Operation: Ammonium Removal Under Increasing Nitrogen Loads

**FIGURE 1:**
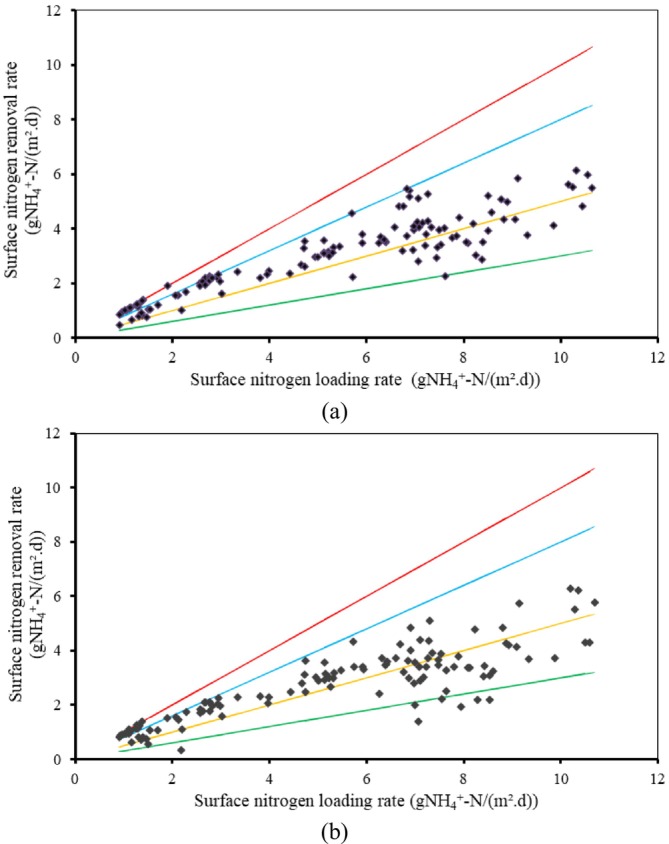
Relationship between surface nitrogen loading rate (SNLR) and surface nitrogen removal rate (SNRR) in R1 (a) and R2 (b) during long‐term operation. Solid lines indicate theoretical removal efficiencies of 30% (green), 50% (yellow), 80% (blue), and 100% (red).

After the inoculation period and stabilization of the biofilm, the reactors (R1 and R2), each filled with a different carrier media, were subjected to a gradual increase in the surface nitrogen loading rate (SNLR), ranging from 0.9 to 11 g NH_4_^+^‐N/(m^2^.d). Figure 1 shows the evolution of the SNLR and surface nitrogen removal rates (SNRR) throughout the experiment. Both reactors exhibited similar behavior at lower loads. At SNLRs between 0.9 and 1.4 g NH_4_^+^‐N/(m^2^.d), ammonium removal remained close to 100%, while between 1.4 and 3 g NH_4_^+^‐N/(m^2^.d), removal efficiencies generally stabilized around 80% in both systems. Within this range, reactor performance was relatively stable. However, above 3 g NH_4_^+^‐N/(m^2^.d), greater fluctuations in system performance were observed. Although both reactors tended toward an average of approximately 50% ammonium removal at SNLRs exceeding 3 g NH_4_^+^‐N/(m^2^.d), the reactor filled with Mutag Biochip media (R2) was generally more affected by the increased nitrogen load, particularly above 6 g NH_4_^+^‐N/(m^2^.d), under which more pronounced performance variations occurred. The average ammonium removal efficiency (%) for each reactor across the evaluated nitrogen loading ranges is presented in Figure S1.

**FIGURE 2:**
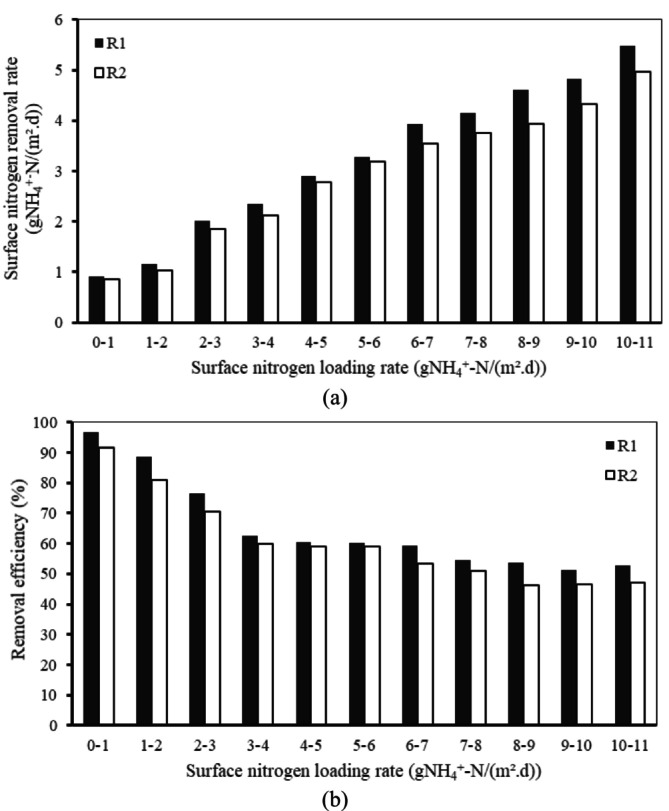
Relationship between surface nitrogen loading rate (SNLR) and surface nitrogen removal rate (SNRR) (a) and ammonia nitrogen removal efficiency (b) for R1 and R2 under increasing nitrogen loads. Bars represent time‐averaged values within each loading range.

To facilitate comparison of the nitrification performance between the reactors, Figure 2a presents the average SNRR for each applied SNLR range, along with the mean corresponding ammonia nitrogen removal efficiency. The results indicate that R2 generally exhibited lower SNRR values than R1 for all SNLR ranges. With the increasing SNLR, a decrease in ammonia removal efficiency was noticed for both systems (Figure 2b). Nevertheless, despite this reduction in efficiency, the SNRR increased, indicating that the reactors were capable of removing more ammonia under higher loading conditions (Figure 2a). However, the maximum SNRR could not be determined, as neither system reached its removal limit within the applied SNLR ranges. For SNLRs between 8–10 g NH_4_^+^‐N/(m^2^.d) for R1 and 7–9 g NH_4_^+^‐N/(m^2^.d) for R2, the SNRR approached apparent plateaus at approximately 4.7 g NH_4_^+^‐N/(m^2^.d) and 3.8 gNH_4_^+^‐N/(m^2^.d), respectively. However, these values do not represent the maximum removal capacity, as stabilization was not maintained at SNLRs above 10 g NH_4_^+^‐N/(m^2^.d) for R1 and 9 g NH_4_^+^‐N/(m^2^.d) for R2, suggesting that the true maximum nitrification capacity was not reached. Additional data on volumetric nitrogen removal rates are provided in Figure S2.

An additional analysis relating surface nitrogen removal rate (SNRR) to effluent (bulk) ammonium concentration for both reactors was performed to provide further insight into the nitrification behavior of the systems under increasing nitrogen loading conditions and to evaluate possible changes in substrate utilization patterns at elevated ammonium concentrations. In both systems, SNRR increased with increasing effluent ammonium concentration, although distinct trends were observed between the carrier media (Figure S3). R1 generally achieved higher SNRR values than R2 at comparable ammonium concentrations, indicating greater overall nitrification capacity. At higher effluent ammonium concentrations, the increase in SNRR became less pronounced, suggesting progressive limitations in nitrification performance under elevated nitrogen loading conditions.

**FIGURE 3:**
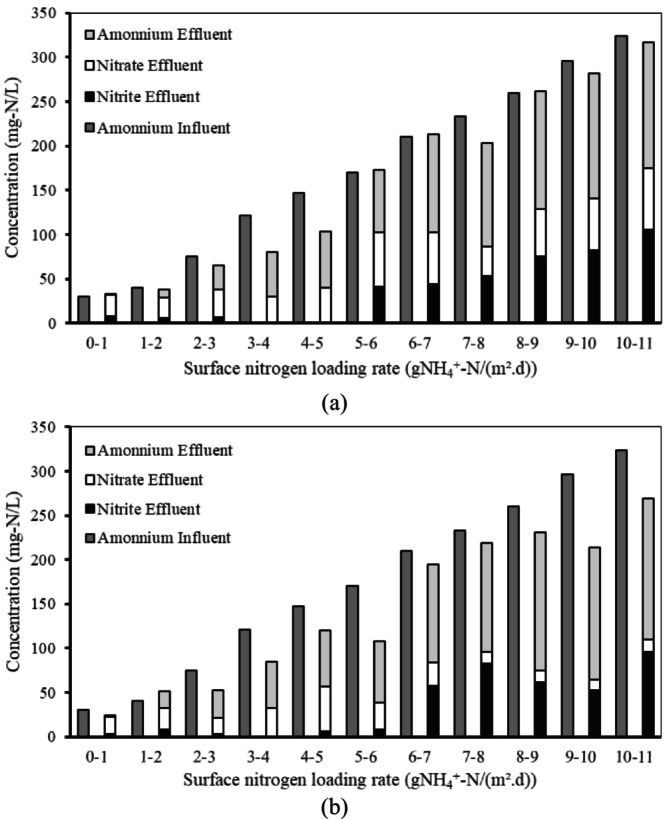
Average influent and effluent concentrations of inorganic nitrogen species (NH_4_^+^, NO_2_^−^ and NO_3_^−^) at different surface nitrogen loading rates in R1 (a) and R2 (b). Values represent time‐averaged data within each loading range.

Figure 3 presents the concentrations of nitrogen species for the applied SNLR ranges. Nitrate predominated in the effluent at SNLRs of 0–7 g NH_4_^+^‐N/(m^2^.d) for R1 and 0–6 g NH_4_^+^‐N/(m^2^.d) for R2, while nitrite was detected only in minor amounts. Notably, for SNLR ranges of 3–4 and 4–5 g NH_4_^+^‐N/(m^2^.d) in R1, and 3–4 g NH_4_^+^‐N/(m^2^.d) in R2, residual ammonium was present in the effluent. Still, nitrite accumulation was negligible, indicating that all removed ammonia was fully converted to nitrate. At higher SNLRs, above 7 g NH_4_^+^‐N/(m^2^.d) for R1 and 6 g NH_4_^+^‐N/(m^2^.d) for R2, partial nitrification was noticed, as indicated by increased nitrite accumulation, particularly in R2.

As there was no organic carbon in the influent stream and no intentional anoxic zones established for denitrification, the observed differences in total nitrogen between influent and effluent (i.e., nitrogen removal from the bulk liquid) are likely due to nitrogen assimilation for microbial growth and potential nitrogen losses as N_2_O emissions during nitrification (Ali et al. 2014; Li et al. 2017).

**FIGURE 4:**
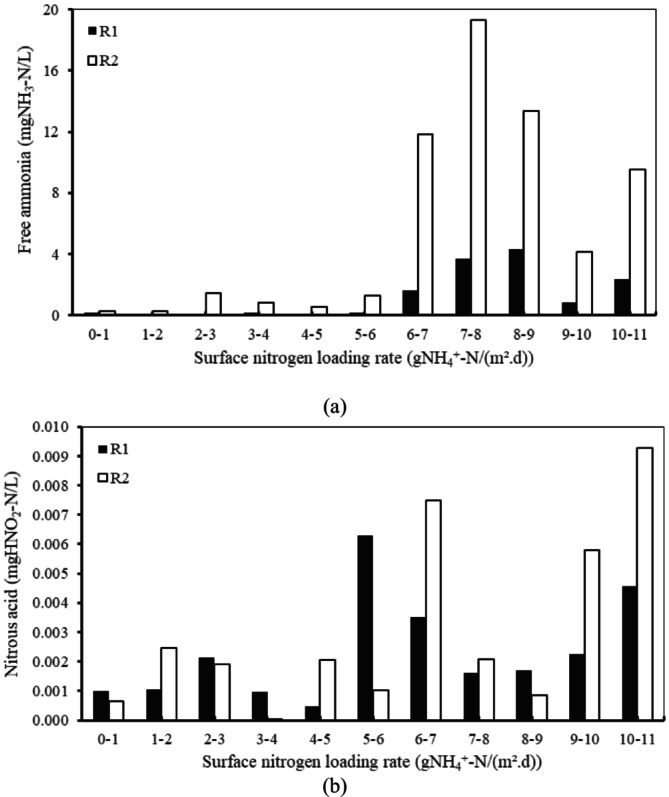
Calculated free ammonia (FA) (a) and free nitrous acid (FNA) (b) concentrations in R1 and R2 at different surface nitrogen loading rates (SNLRs). Values were estimated based on measured pH, temperature, and nitrogen species concentrations.

Nitrification performance can be affected by the presence of inhibitory compounds, such as FA and FNA. The concentrations of these non‐ionized species depend on the total ammonium or nitrite contents, as well as on pH and temperature, which influence the equilibrium between the ionized and non‐ionized forms (see Section 2.2). The estimated concentration of FA and FNA in each reactor is displayed in Figure 4. It can be observed that for all evaluated nitrogen loads, FA content was higher in R2, while in R1 it was only present for SNLRs above 6 g NH_4_^+^‐N/(m^2^.d) (Figure 4a). Up to 6 g NH_4_^+^‐N/(m^2^.d), FA concentration in R2 was always below 1.4 mg NH_3_‐N/L, while for SNLRs above 6 g NH_4_^+^‐N/(m^2^.d), its concentration increased significantly, reaching its highest value (19 mg NH_3_‐N/L) for SNLRs between 7 and 8 g NH_4_^+^‐N/(m^2^.d). On the other hand, the highest concentration of FA found in R1 was 4.3 mg NH_3_‐N/L, when the SNLR was between 8 and 9 g NH_4_^+^‐N/(m^2^.d). Despite the accumulation of nitrite under some loads, the estimated FNA concentration was low in both systems (Figure 4b), not exceeding 0.009 mg HNO_2_‐N/L.

Data on the inlet and outlet pH of the systems are presented in Figure S4. It can be observed that acidification of the medium occurred in all analyzed experimental conditions. The pH drop was more pronounced in R1, mainly between SNLRs 3 to 6 and 9 to 10 g NH_4_^+^‐N/(m^2^.d). The largest drop occurred in the SNLR range of 4 to 5 g NH_4_^+^‐N/(m^2^.d), when the pH reached 5.9. R2 showed greater pH stability, with values above 6.8.

### 3.2 Suspended and Adhered Biomass Fractions

**FIGURE 5:**
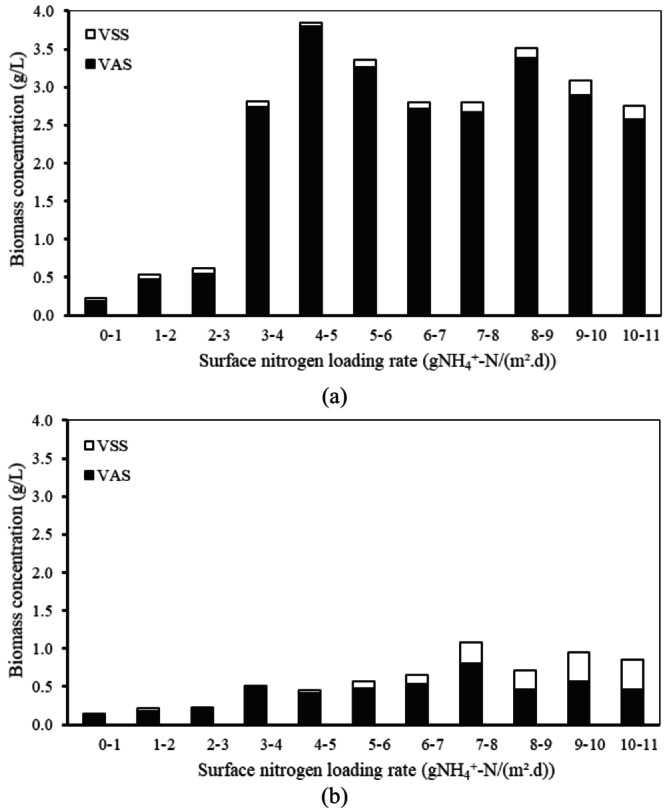
Volatile attached solids (VAS) and volatile suspended solids (VSS) at different surface nitrogen loading rates in R1 (a) and R2 (b).

The dynamics of suspended and attached volatile biomass for all surface nitrogen loading rate ranges are shown in Figure 5. To facilitate comparison, both biomass fractions were expressed in g/L. In R1, the attached biomass concentration increased markedly from 0.2 to 3.8 g VAS/L as the nitrogen load rose from 0 to 5 g NH_4_^+^‐N/(m^2^.d) (Figure 5a), indicating enhanced microbial attachment and biofilm development under moderate loading. However, at higher loading ranges (5–11 g NH_4_^+^‐N/(m^2^.d)), this trend was not maintained. A decrease in attached biomass (from 3.8 to 2.7 g VAS/L) was observed between 5 and 8 g NH_4_^+^‐N/(m^2^.d), followed by a slight recovery (to 3.4 g VAS/L) at 8–9 g NH_4_^+^‐N/(m^2^.d) and another decline (to 2.6 g VAS/L) at 9–11 g NH_4_^+^‐N/(m^2^.d). This transient recovery at intermediate‐high loads may reflect short‐term microbial adaptation and compensatory growth of tolerant nitrifying populations, partially offsetting biomass losses. However, this effect was not sustained under further load increase, indicating progressive stress and structural instability of the biofilm. These oscillations likely reflect cycles of biofilm thickening, partial detachment, and structural reorganization under increasing nitrogen loads and elevated FA concentrations. The transient recovery observed at intermediate‐high loads (8–9 g NH_4_^+^‐N/(m^2^.d)) suggests adaptive regrowth of active biomass following detachment events, rather than sustained inhibition. At the highest loads, further biomass loss indicates that detachment and growth limitations prevailed over biofilm stabilization.

Regarding suspended biomass in R1, a very low concentration was observed for all experimental conditions when compared to attached biomass, corresponding to less than 20% of the total volatile solids (VAS + VSS) (Figure S5). There was a gradual increase in the planktonic biomass fraction as the SNLR was increased, ranging from 0.05 to 0.19 gVSS/L, suggesting increased biomass detachment under higher loads.

Although the reactor filling fraction was chosen so that the surface area for biofilm growth was the same in both systems, the average VAS content on the Mutag Biochip media in R2 was only 0.5 g/L (Figure 5b). Furthermore, unlike what was observed in R1, the solids adhered to the media in R2 increased slowly as the surface nitrogen loading rate was increased from 0 to 8 g NH_4_^+^‐N/(m^2^.d), reaching a maximum concentration of 0.8 g/L. From 8 g NH_4_^+^‐N/(m^2^.d) onwards, there was a decrease in adhered volatile biomass concentration. Overall, R2 exhibited consistently low attached biomass concentrations, confirming limited biofilm accumulation and higher susceptibility to shear‐induced detachment.

Regarding VSS in the reactor filled with Mutag Biochip media, very low concentrations can be observed for SNLRs lower than 5 g NH_4_^+^‐N/(m^2^.d), corresponding to less than 15% of the total volatile solids (VAS + VSS) (Figure S4). However, the fraction of suspended solids increased, corresponding to more than 40% of the total volatile solids for SNLRs between 5 and 11 g NH_4_^+^‐N/(m^2^.d). This increase in suspended solids suggests enhanced biomass detachment from the carrier media under elevated nitrogen loading conditions, particularly in R2.

**FIGURE 6:**
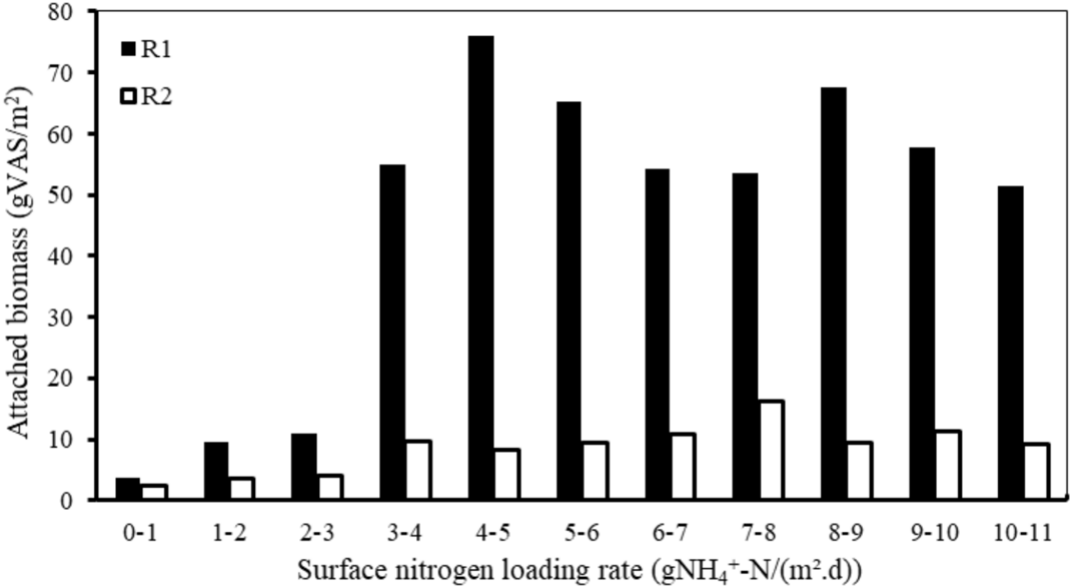
Attached biomass per unit surface area (g VAS/m^2^) for R1 and R2 at different surface nitrogen loading rates.

The comparison between the amount of immobilized biomass per area (m^2^) of carrier for the two systems is illustrated in Figure 6. For both R1 and R2, an increase in biomass adhesion was observed at lower SNLR ranges, and then tended to stabilize for loading rates above 4 g NH_4_^+^‐N/(m^2^.d). Kaldnes K1 media (R1) presented a higher biomass content and also greater oscillation than the Mutag Biochip carriers (R2). The average surface biomass content in R1 (approximately 60 gVAS/m^2^) was approximately 4.5 times greater than in R2 (approximately 13 gVAS/m^2^). The inert material (fixed solids) in the biofilm of R1 and R2 represented only approximately 25% and 20% of the TAS, respectively.

### 3.3 Apparent Biomass‐Specific Ammonium Removal Rate

**FIGURE 7:**
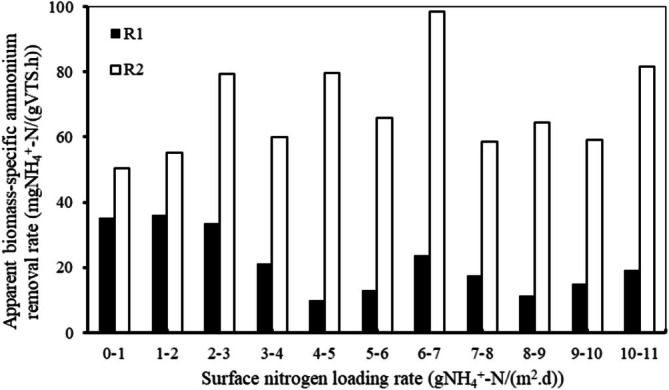
Apparent biomass‐specific ammonium removal rate R1 and R2 at different surface nitrogen loading rates.

Figure 7 illustrates the average biomass‐specific ammonium removal rate in each SNLR range. It can be observed that, for all evaluated loads, the apparent biomass‐specific ammonium removal rate of R2 tended to be higher than that of R1. This behavior indicates that, despite exposure to higher FA levels, the remaining active biomass in R2 maintained elevated specific activity, likely due to reduced diffusion limitations associated with the thinner biofilm established in the Mutag Biochip media. Initially, at SNLR between 0 and 3 g NH_4_^+^‐N/(m^2^.d) for R1 and 0 to 2 g NH_4_^+^‐N/(m^2^.d) for R2, a relatively constant apparent biomass‐specific ammonium removal rate was observed in both systems, reaching approximately 38 mg NH_4_^+^‐N/(gVTS.h) in R1 and 52 mg NH_4_^+^‐N/(g VTS.h) in R2.

As the nitrogen load gradually increased and biomass accumulated in the carrier media, oscillations in the apparent biomass‐specific ammonium removal rate were observed in both systems. However, the apparent activity exhibited distinct behaviors between the reactors. In R1, for SNLRs above 4 g NH_4_^+^‐N/(m^2^.d), the biomass showed lower nitrifying activity compared to that at lower loads, suggesting possible inhibitory effects or biofilm limitations at higher nitrogen inputs. In contrast, an increase in apparent biomass‐specific ammonium removal rate with higher loads was observed in R2, reaching 98 mg NH_4_^+^‐N/(g VTS.h) at an SNLR range of 6–7 g NH_4_^+^‐N/(m^2^.d).

## 4 Discussion

### 4.1 Effect of Carrier Type on Biomass Dynamics at Increasing Nitrogen Loading Rates

The effective surface area available for microbial attachment is a key design parameter in MBBR systems (Ødegaard et al. 2000; Deena et al. 2022). However, several studies have emphasized that system performance depends more strongly on the applied surface loads than on the nominal surface area or material of the carrier (Forrest et al. 2016; Ødegaard et al. 2000; Young et al. 2016). In this study, the two reactors (R1 and R2) were designed to provide identical theoretical surface areas for biofilm growth (250 m^2^/m^3^) by adjusting the filling fraction of each carrier type. Consequently, both reactors were expected to provide comparable theoretical capacities for biofilm development based on their equivalent nominal surface areas.

Nevertheless, the results revealed a substantially higher attached biomass content in R1 (filled with Kaldnes K1 media) compared with R2 (containing Mutag Biochip media), despite equivalent theoretical surface areas and identical operating conditions (aeration intensity, hydraulic retention time, and influent composition).

The higher biomass retention in R1 may be associated with the geometry and mechanical stability of the Kaldnes K1 carriers. Their hollow cylindrical shape provides a protected internal cavity, which may reduce shear stress and inter‐particle collisions, enabling the formation of a thick and cohesive biofilm. Similar effects of media geometry and media dimensions on biofilm accumulation and nitrification efficiency have been reported, with longer and protected carrier structures promoting thicker biofilms and enhanced nitrifying activity relative to simpler media configurations (Garcia et al. 2022). In contrast, the parabolic and porous design of Mutag Biochip media exposes the biofilm to higher shear caused by vigorous carrier motion.

These mechanical effects may promote more frequent biofilm sloughing, which could contribute to the lower attached biomass and higher suspended solids observed in R2 under high nitrogen loads. Additionally, biomass accumulation within the porous structure of Mutag Biochip media may progressively reduce surface accessibility and partially obstruct pore channels, limiting the effective area available for active biofilm growth over time (Forrest 2014). The Kaldnes K1 carriers, being non‐porous, are less prone to such events, maintaining a stable, effective surface area over time. This may help explain the higher VAS concentrations measured in R1 (average≈3.4 g VAS/L) compared with R2 (average≈0.8 g VAS/L), as well as the larger surface‐specific biomass content in R1 (~60 g VAS/m^2^ versus 13 g VAS/m^2^ for R2).

Regarding the suspended biomass fraction, R1 consistently presented lower VSS concentrations, within the 150–250 g/m^3^ range reported by Ødegaard et al. (2010) as typical for MBBRs. In contrast, R2 exhibited substantially higher VSS concentrations (up to 400 g SS/m^3^) under high SNLRs (9–11 g NH_4_^+^‐N/(m^2^.d)), indicating increased biomass detachment and possibly hindered biofilm development due to pore saturation. This behavior suggests that media configuration, in addition to nominal surface area, may influence the partitioning between attached and suspended biomass and, consequently, reactor stability under elevated nitrogen loads.

### 4.2 Effect of Increasing Nitrogen Loading Rates and Carrier Type on Nitrification and Apparent Biomass‐Specific Ammonium Removal Rate

The results indicate that both MBBR systems, despite being filled with distinct carrier media, maintained effective ammonium removal under progressively increasing surface nitrogen loading rates (SNLRs), with average efficiencies near 50%. This behavior is consistent with the robustness of MBBR systems under sustained high‐loading conditions, a characteristic well documented for organic loads (Bassin et al. 2016; Lima et al. 2016; Young et al. 2017). However, few studies have focused on the behavior of MBBRs subjected to high nitrogen loads, particularly above 3 g NH_4_^+^‐N/(m^2^.d), and the corresponding influence of carrier type on nitrification performance. Providing insights into the limits of nitrification capacity and the role of media configuration under high nitrogen load conditions is important from an operational and design perspective, as it supports the optimization of MBBR systems for stable and efficient nitrogen removal in high‐strength wastewaters.

Although prior research suggests that the type of carrier may have a limited influence on system performance for organic carbon removal (Bassin et al. 2016; Forrest et al. 2016; Young et al. 2016), our findings suggest that the carrier configuration may influence nitrification performance when nitrogen loads become elevated. Above 4 g NH_4_^+^‐N/(m^2^.d), clear divergences between the two reactors were observed. The reactor filled with Kaldnes K1 media (R1) exhibited higher and more stable ammonium removal rates, while the Mutag Biochip system (R2) experienced greater performance variability as ammonium load increased. These differences became especially apparent at SNLRs above 6 g NH_4_^+^‐N/(m^2^.d), when notable nitrite accumulation and reduced total ammonium oxidation were observed in R2. The observed nitrite buildup in R2 may be associated with the inhibitory effects of FA on NOB, which are widely documented to be more sensitive to FA than AOB. Recent work has confirmed that higher FA concentrations may suppress NOB activity, leading to sustained nitrite accumulation in aerobic biofilm systems under high ammonia loads (Jeong et al. 2024). The increase in FA concentration above 6 g NH_4_^+^‐N/(m^2^.d) reached levels up to 19 mg NH_3_–N/L that are within the range reported to exceed the tolerance threshold for NOB. This is consistent with studies demonstrating that NOB are inhibited by FA at concentrations as low as 1 mg NH_3_–N/L, with significant growth suppression above 6 mg NH_3_–N/L (Vadivelu et al. 2007). Park and Bae (2009) also reported a low inhibition constant for FA in NOB, confirming their vulnerability compared with AOB. Consequently, the elevated FA concentrations observed in R2 under high load conditions may impair nitrite oxidation, leading to partial nitrification and higher nitrite accumulation, as observed in this study. However, the distinct responses observed between the reactors suggest that carrier‐related biomass retention characteristics may also have influenced the resilience of the systems to increasing FA concentrations.

In addition to FA‐related effects, the contrasting performance observed between the reactors may also be associated with differences in biomass retention and biofilm stability between the two carrier types. R1 maintained higher attached biomass concentrations throughout the study, whereas R2 exhibited lower biomass retention and higher suspended solids under elevated nitrogen loads.

The difference in biofilm distribution between the two media also affects substrate and oxygen diffusion. In R1, the thick biofilm growing within the internal cavities of Kaldnes K1 may experience substrate and oxygen diffusion limitations that reduce the activity of the innermost layer. This suggests that part of the biomass may have been less accessible for nitrification. However, the larger quantity of immobilized biomass may provide a sufficient active zone near the biofilm surface to sustain nitrification. In contrast, the thin configuration of the Mutag Biochip media limits biofilm thickness while exposing the biofilm to substantial frictional and shear forces during mixing, which may promote frequent detachment of the outer layers and reduce the overall amount of immobilized biomass. Such carrier configuration may result in smaller diffusion distances, which may enhance the activity of the attached biomass, partially compensating for inhibitory effects associated with elevated FA concentrations. This mechanism is consistent with the higher apparent biomass‐specific ammonium removal rate observed in R2 (up to 98 mg NH_4_^+^‐N/(g VTS.h)) compared with R1 (up to 35 mg NH_4_^+^‐N/(g VTS.h)). Nevertheless, this higher specific activity was insufficient to compensate for the lower total biomass retention, resulting in a smaller overall nitrogen removal rate.

The maximum SNRR values observed in the present study (approximately 5.0 g NH_4_^+^‐N/(m^2^.d) in R1 and 4.5 g NH_4_^+^‐N/(m^2^.d) in R2) (Figure S3) were comparable to values reported for high‐rate nitrifying biofilm systems treating ammonium‐rich wastewaters (Niu et al. 2024; Jiang et al. 2020). Previous studies have shown that carrier configuration may have a limited influence on nitrification performance under moderate loading conditions, but its importance becomes more evident at elevated nitrogen loads due to differences in biofilm thickness, biomass retention, clogging potential, detachment dynamics, and biomass distribution (Young et al. 2016). In long‐term MBBR operation with Kaldnes K1 and Mutag Biochip carriers, increasing loading rates have also been associated with changes in biofilm accumulation and the relative contribution of attached and suspended biomass to nitrification performance (Bassin et al. 2016). These observations are consistent with the higher biomass inventory and more stable nitrification performance observed in R1, further supporting the view that biofilm retention characteristics, in addition to nominal surface area, are important determinants of reactor performance under elevated nitrogen loading conditions (Young et al. 2016; Niavol et al. 2025). The additional analysis relating SNRR to effluent ammonium concentration further supported the distinct nitrification behaviors observed between the reactors. R1 generally maintained higher SNRR values over a broader range of effluent ammonium concentrations, whereas R2 exhibited a less pronounced increase in SNRR at elevated ammonium concentrations. This behavior suggests that nitrification performance in R2 became progressively limited under higher nitrogen loading conditions, despite the relatively high apparent biomass‐specific activity. These results are consistent with the lower biomass retention and the distinct transport characteristics associated with the Mutag Biochip media.

Overall, the nitrification performance observed in this study under high nitrogen loading appeared to be influenced by both operational factors, particularly FA accumulation under elevated SNLRs, and carrier‐related characteristics affecting biofilm retention, shear exposure, and substrate transport. The findings suggest that biofilm retention stability may have had a stronger influence on nitrification performance than nominal surface area alone under the evaluated conditions, particularly under conditions of elevated FA concentrations. While surface area defines the potential for microbial attachment, the amount of active and well‐retained biomass appears to have influenced reactor performance under elevated nitrogen loads. From a practical perspective, these findings suggest that carrier selection for high‐ammonium wastewaters should consider not only nominal surface area, but also the ability of the media to promote stable biofilm retention under elevated nitrogen loading conditions. Notably, these trends emerged only after prolonged operation under progressively increasing nitrogen loads, highlighting the relevance of long‐term monitoring for identifying stability thresholds and resilience patterns in MBBR systems.

It should be noted that the present study evaluated only two carrier configurations under laboratory‐scale and strictly autotrophic conditions. Therefore, the observed trends should not be generalized to all carrier types or operational scenarios. Further investigations involving additional media geometries, scales, and wastewater compositions are necessary to confirm the broader applicability of these findings.

## 5 Conclusions

This study evaluated the effects of increasing nitrogen loading rates and carrier type on nitrification performance, nitrifying activity, and biomass behavior in two moving bed biofilm reactors (MBBRs), R1 and R2. The results obtained under the evaluated conditions suggest that nitrification performance was influenced not only by nominal surface area but also by carrier‐related biofilm retention characteristics. Kaldnes K1 media (used in R1), characterized by a non‐porous and protected geometry, favored the formation of thicker and more stable biofilms, providing higher resistance to FA inhibition and operational fluctuations. In contrast, the Mutag Biochip used in R2, despite being added to have the same theoretical surface area as R1, exhibited limited biofilm retention due to detachment under high shear caused by its intrinsic configuration, resulting in lower biomass accumulation and greater performance variability. These findings suggest that nitrification performance depends on the interplay between biofilm accumulation and apparent ammonium removal capacity, all influenced by carrier configuration. From a process‐engineering perspective, the results suggest that biofilm retention capacity and reactor stability should be considered alongside nominal surface area during carrier selection for high‐ammonium wastewater treatment. Under the evaluated conditions, performance differences between the carriers became particularly evident at surface nitrogen loading rates above 6 g NH_4_^+^‐N/(m^2^.d), when increased nitrite accumulation and higher FA concentrations were observed, particularly in R2. Overall, this study contributes to the understanding of nitrification limits in MBBRs and provides practical guidance for designing resilient, high‐performance biofilm systems for treating high‐strength nitrogen wastewaters. Further studies involving additional carrier types, operational conditions, and wastewater compositions are still necessary to confirm these trends under a broader range of scenarios.

## Author Contributions

**Isabelli Dias Bassin:** conceptualization, investigation, supervision, project administration, writing – original draft, writing – review and editing, funding acquisition, validation, methodology, data curation, formal analysis. **Renato Rocha Valerio:** methodology, investigation, formal analysis. **João Paulo Bassin:** conceptualization, investigation, project administration, supervision, writing – review and editing, resources, funding acquisition, validation, methodology, data curation, formal analysis.

## Funding

This work was supported by the Coordenação de Aperfeiçoamento de Pessoal de Nível Superior, the Conselho Nacional de Desenvolvimento Científico e Tecnológico, and the Fundação Carlos Chagas Filho de Amparo à Pesquisa do Estado do Rio de Janeiro.

## Conflicts of Interest

The authors declare no conflicts of interest.

## Supporting information


**Table S1:** Surface nitrogen loading rate (SNLR) and surface nitrogen removal rate (SNRR) in different reactor systems under diverse influent organic matter (COD or BOD) concentrations.
**Table S2:** Main characteristics of the carrier media used in the MBBR systems.
**Figure S1:** Performance of each system for the analyzed surface nitrogen loading rate ranges. R1(a), R2 (b).
**Figure S2:** Volumetric nitrogen removal rate as a function of the volumetric nitrogen loading rate.
**Figure S3:** Relationship between surface nitrogen removal rate (SNRR) and effluent ammonium concentration in R1 and R2 under increasing surface nitrogen loading conditions. Values represent time‐averaged data obtained for each evaluated SNLR range.
**Figure S4:** pH variation at different surface nitrogen loading rates in R1(a) and R2 (b). Black bars represent pH in the influent, and gray bars represent pH in the outlet stream.
**Figure S5:** Contribution of attached and suspended biomass in R (a) and R2 (b). Black bars represent attached biomass, and white bars represent suspended biomass.

## Data Availability

The data that support the findings of this study are available from the corresponding author upon reasonable request.
