# Supplementary material for: Beyond Surface Area: The Role of Carrier Media Structure on Nitrification Performance and Biomass Activity in MBBR Systems Under High Nitrogen Loads
Source: Water Environ Res. 2026 Jul 16;98(7):e70495. doi: 10.1002/wer.70495 (PMC13376700; doi:10.1002/wer.70495)
Supplement: Supplementary file 1 — Table S1: Surface nitrogen loading rate (SNLR) and surface nitrogen removal rate (SNRR) in different reactor systems under diverse influent organic matter (COD or BOD) concentrations. Table S2: Main characteristics of the carrier media used in the MBBR systems. Figure S1: Performance of each system for the analyzed surface nitrogen loading rate ranges. R1(a), R2 (b). Figure S2: Volumetric nitrogen removal rate as a function of the volumetric nitrogen loading rate. Figure S3: Relationship between surface nitrogen removal rate (SNRR) and effluent ammonium concentration in R1 and R2 under increasing surface nitrogen loading conditions. Values represent time‐averaged data obtained for each evaluated SNLR range. Figure S4: pH variation at different surface nitrogen loading rates in R1(a) and R2 (b). Black bars represent pH in the influent, and gray bars represent pH in the outlet stream. Figure S5: Contribution of attached and suspended biomass in R (a) and R2 (b). Black bars represent attached biomass, and white bars represent suspended biomass. [file WER-98-e70495-s001.docx]

Supplementary Material for: **Beyond Surface Area: The Role of Carrier Media Structure on Nitrification Performance and Biomass Activity in MBBR Systems Under High Nitrogen Loads**

Isabelli Dias Bassin^a^, Renato Rocha Valerio^b^; João Paulo Bassin^b,c*^

^a^Department Department of Biochemical Engineering, School of Chemistry, Federal University of Rio de Janeiro, Rio de Janeiro, Brazil

^b^Chemical Engineering Program, COPPE, Federal University of Rio de Janeiro, Rio de Janeiro, RJ, Brazil

^c^Civil Engineering Program, COPPE, Federal University of Rio de Janeiro, Rio de Janeiro, RJ, Brazil

* Corresponding author. Mailing address: Chemical Engineering Program/COPPE, Federal University of Rio de Janeiro, P.O. Box 68502, 21941-972, Rio de Janeiro, Brazil, Email address: jbassin@peq.coppe.ufrj.br

**S.1 Supplementary Information**

*S.1.1* *Free ammonia (FA) and free nitrous acid (FNA) concentrations*

To determine the concentration of free ammonia (NH_3_-N) in the nitrifying reactors, Equations (S1 and S2), proposed by Anthoniesen et al. (1976), were used. These equations take into account the equilibrium between the ionized and non-ionized forms of ammoniacal-N, in addition to the pH and T of the medium.

$[NH_{3}-N]_{i}=\frac{[{NH}_{4}^{+}-N]_{ef-i} x 10pH_{i}}{\frac{1}{Ka_{i}}+10pH_{i}}$ (S1)

$\frac{1}{Ka_{i}}=\exp\left( \frac{6334}{273.15+T_{i}} \right)$ (S2)

Where

[NH_3_-N] is the concentration of free ammonia (NH_3_-N) in mg NH_3_-N/L,

[NH_4_^+^-N]_ef-i_ is the concentration of NH_4_^+^-N in mg NH_4_^+^-N/L,

pH is the hydrogen potential,

T is the temperature in ºC, and the index i represents the reactor analyzed.

Due to the possibility of intensification in the generation and emission of N_2_O in systems with high nitrous acid (HNO_2_) contents, the determination of this compound was also carried out as a function of the NO_2_^-^-N concentrations of the system, the pH and T. Equations (S3) and (S4), also suggested by Anthoniesen et al. (1976), were used.

$[HNO_{2}-N]_{i}=\frac{[{NO}_{2}^{-}-N]_{i}}{Kn_{i} x 10pH_{i}}$ (S3)

$Kn_{i}=\exp\left( \frac{-2300}{273.15+T_{i}} \right)$ (S4)

Where

[HNO_2_-N] is the concentration of nitrous acid (HNO_2_-N) in mg HNO_2_-N/L,

[NO_2_^-^-N] is the concentration of NO_2_^-^-N in mg NO_2_^-^-N/L,

pH is the hydrogen potential,

T is the temperature in °C, and the index i represents the reactor analyzed.

**S.2 Supplementary Tables**

Table S1: Surface nitrogen loading rate (SNLR) and surface nitrogen removal rate (SNRR) in different reactor systems under diverse influent organic matter (COD or BOD) concentrations.

| **Reactor**  **system** | **SNLR**  **(gN** **NH_4_^+^–N /(m^2^.d))** | **SNRR**  **(gN** **NH_4_^+^–N /(m^2^.d))** | **Influent**  **(COD or BOD)** | **Reference** |
| --- | --- | --- | --- | --- |
| MBBR | 0.03 - 0.50 | 0.02 - 0.45 | 3.8 - 4.7mg sCOD/L | Zhang et al. (2013) |
| MBBR | 0.90 - 1.9 | 0.80 - 1.88 | 5 mg BOD_5_/L | Forrest et al. (2016) |
| MBBR | 1.89 | 1.82 | 10 mg BOD_5_/L | Young et al. (2016) |
| MBBR | 1.60 | 0.90 | 400 mg sCOD/L | Bassin et al. (2016) |
| MBBR | 1.30 - 2.30 | 1.27 - 2.13 | 32 mg sCOD/L | Young et al. (2017a) |
| MBBR | 2.36 | 2.00 | 55 mg sCOD/L | Young et al. (2017b) |
| MBBR |  | 0.88 | 10 mg COD/L | Ren et al. (2024) |

Table S2. Main characteristics of the carrier media used in the MBBR systems.

| **Characteristic** | **AnoxKaldnes K1 (R1)** | **Mutag Biochip (R2)** |
| --- | --- | --- |
| Material | High-density polyethylene | High-density polyethylene |
| Structure | Non-porous, hollow cylindrical | Porous, flat disk |
| Nominal specific surface area | 500 m^2^/m^3^ | 3000 m^2^/m^3^ |
| Nominal diameter (mm) | 9.1 | 22 |
| Nominal length/thickness (mm) | 7.2 | 0.8 – 1.2 |
| Density | ~0.95 g/cm^3^ | ~0.95 g/cm^3^ |
| Biofilm retention mechanism | Surface attachment within the protected area | Surface and pore colonization |
| Filling fraction in this study | 50% | 8,3% |
| Approximate number of carrier units in reactor | 100 | 29 |
| Effective specific surface area in the reactor | 250 m^2^/m^3^ | 250 m^2^/m^3^ |
| Representative image | 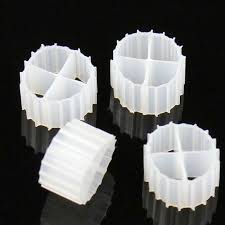 | 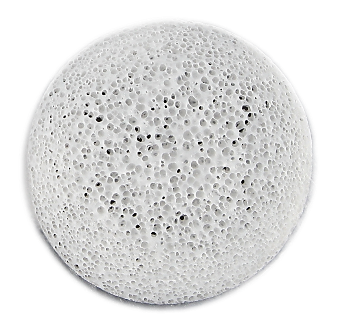 |

**S.3 Supplementary Figures**

| 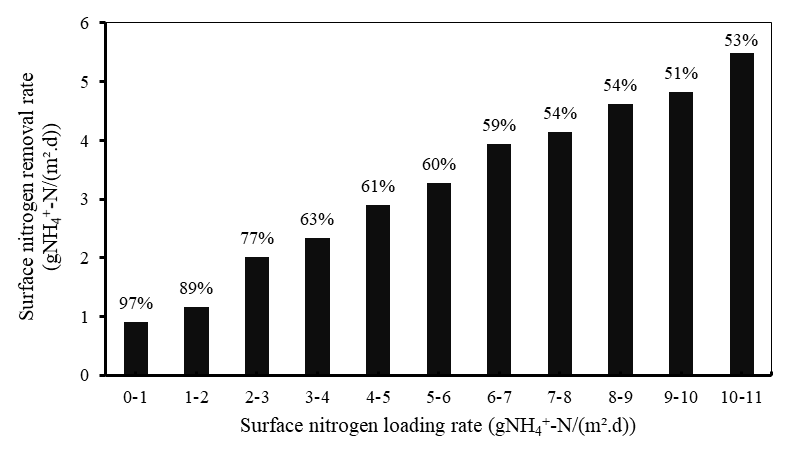 |
| --- |
| (a) |
| 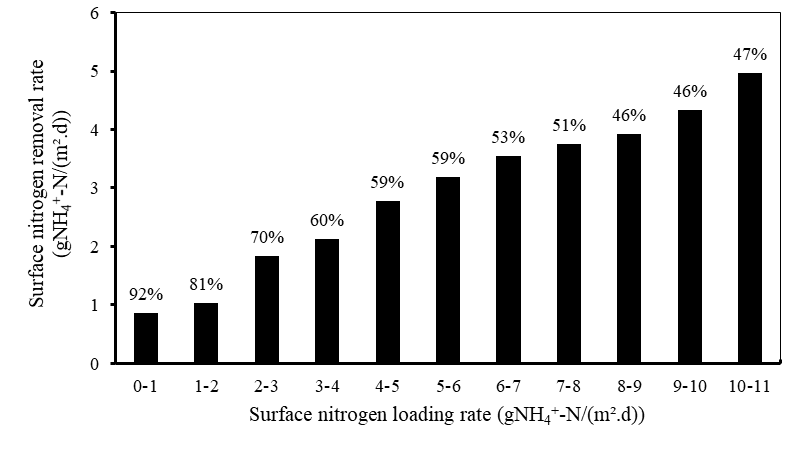 |
| (b) |

Figure S1. Performance of each system for the analyzed surface nitrogen loading rate ranges. R1(a), R2 (b).


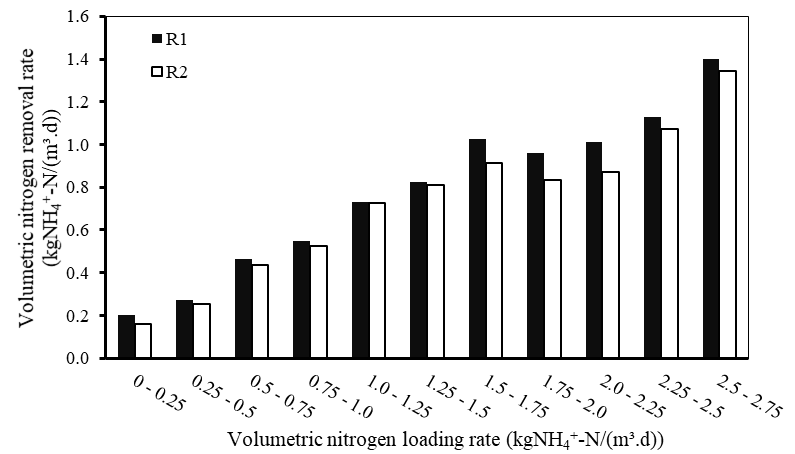


Figure S2. Volumetric nitrogen removal rate as a function of the volumetric nitrogen loading rate.

Figure S3. Relationship between surface nitrogen removal rate (SNRR) and effluent ammonium concentration in R1 and R2 under increasing surface nitrogen loading conditions. Values represent time-averaged data obtained for each evaluated SNLR range.

| 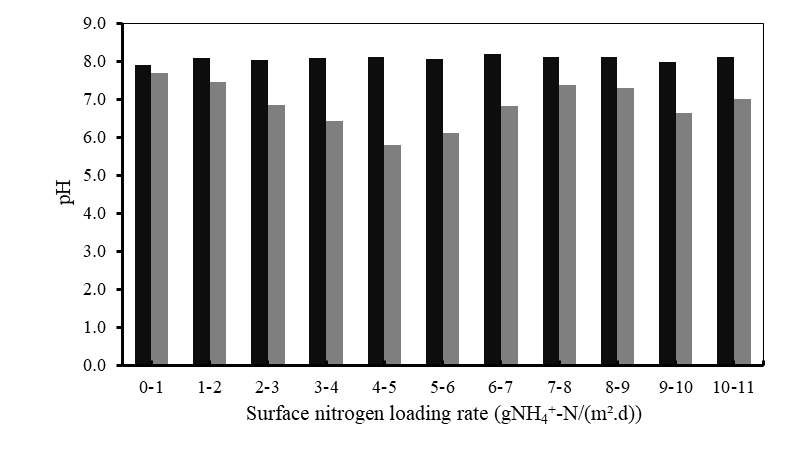 |
| --- |
| (a) |
| 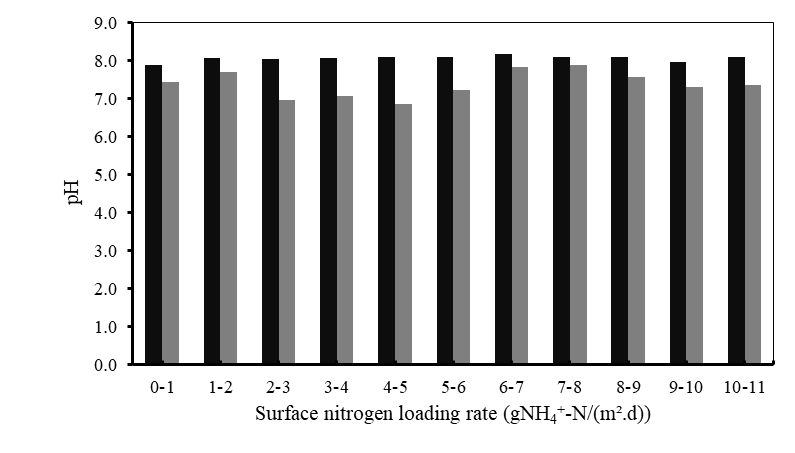 |
| (b) |

Figure S4. pH variation at different surface nitrogen loading rates in R1(a) and R2 (b). Black bars represent pH in the influent, and gray bars represent pH in the outlet stream.

| 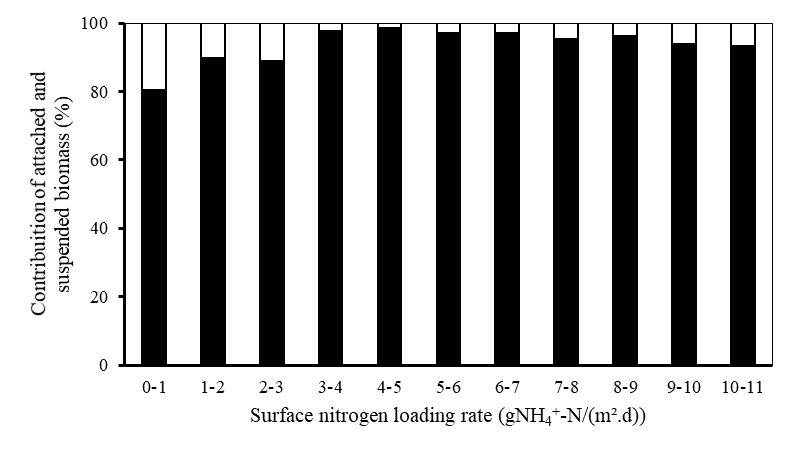 |
| --- |
| (a) |
| 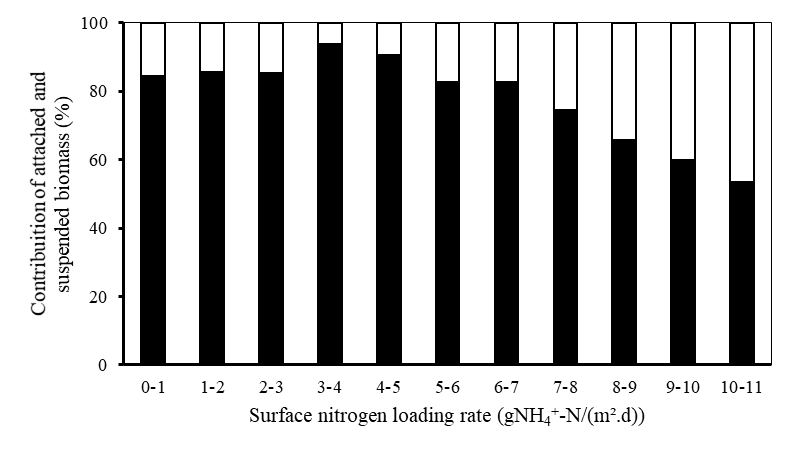 |
| (b) |

Figure S5. Contribution of attached and suspended biomass in R (a) and R2 (b). Black bars represent attached biomass, and white bars represent suspended biomass.

**References**

Anthonisen, A.C., Loehr, R.C., Prakasam, T.B.S. And Srinath, E.G., 1976. Inhibition of nitrification by ammonia and nitrous acid. Journal of the Water Pollution
Control Federation, pp. 835-852.
